# The Complete Chloroplast Genomes of Three *Cardiocrinum* (Liliaceae) Species: Comparative Genomic and Phylogenetic Analyses

**DOI:** 10.3389/fpls.2016.02054

**Published:** 2017-01-10

**Authors:** Rui-Sen Lu, Pan Li, Ying-Xiong Qiu

**Affiliations:** Key Laboratory of Conservation Biology for Endangered Wildlife of the Ministry of Education, College of Life Sciences, Zhejiang UniversityHangzhou, China

**Keywords:** Liliaceae, *Cardiocrinum*, chloroplast genome, genomic structure, phylogenomics, taxonomic identification

## Abstract

The genus *Cardiocrinum* (Endlicher) Lindley (Liliaceae) comprises three herbaceous perennial species that are distributed in East Asian temperate-deciduous forests. Although all three *Cardiocrinum* species have horticultural and medical uses, studies related to species identification and molecular phylogenetic analysis of this genus have not been reported. Here, we report the complete chloroplast (cp) sequences of each *Cardiocrinum* species using Illumina paired-end sequencing technology. The cp genomes of *C. giganteum, C. cathayanum*, and *C. cordatum* were found to be 152,653, 152,415, and 152,410 bp in length, respectively, including a pair of inverted repeat (IR) regions (26,364–26,500 bp) separated by a large single-copy (LSC) region (82,186–82,368 bp) and a small single-copy (SSC) region (17,309–17,344 bp). Each cp genome contained the same 112 unique genes consisting of 30 transfer RNA genes, 4 ribosomal RNA genes, and 78 protein-coding genes. Gene content, gene order, AT content, and IR/SC boundary structures were almost the same among the three *Cardiocrinum* cp genomes, yet their lengths varied due to contraction/expansion of the IR/SC borders. Simple sequence repeat (SSR) analysis further indicated the richest SSRs in these cp genomes to be A/T mononucleotides. A total of 45, 57, and 45 repeats were identified in *C. giganteum, C. cathayanum*, and *C. cordatum*, respectively. Six cpDNA markers (*rps19, rpoC2-rpoC1, trnS-psbZ, trnM-atpE, psaC-ndhE, ycf15-ycf1*) with the percentage of variable sites higher than 0.95% were identified. Phylogenomic analyses of the complete cp genomes and 74 protein-coding genes strongly supported the monophyly of *Cardiocrinum* and a sister relationship between *C. cathayanum* and *C. cordatum*. The availability of these cp genomes provides valuable genetic information for further population genetics and phylogeography studies on *Cardiocrinum*.

## Introduction

The tribe Lilieae sensu Tamura (1998) belongs to Liliaceae sensu APG III (Angiosperm Phylogeny Group, 2009), and contains five genera: *Lilium* L., *Nomocharis* Franch., *Fritillaria* L., *Notholirion* Wallich ex Boissier, and *Cardiocrinum* (Endlicher) Lindley (Gao et al., 2012). This tribe is characterized by papillose tepals (except *Fritillaria*) and numerous fleshy bulb-scales, as well as a morphologically distinct karyotype (Tamura, 1998). Among the five genera, *Cardiocrinum*, the subject of our study, is a small genus of bulbous plants, comprising three species: *C*. *giganteum* (Wall.) Makino, *C. cathayanum* (E. H. Wilson) Stearn, and *C. cordatum* (Thunb.) Makino. These species are long-lived, monocarpic, perennial herbs of East Asian temperate broad-leaved deciduous forests, and mainly differ in individual height, manner of flowering, floral characteristics (e.g., flower number/size/shape, bracts caducous vs. persistent) and geographic distribution (Ohara et al., 2006). Two of them, *C. giganteum* and *C. cathayanum*, form a parapatric species pair with abutting ranges in central China. The former is scattered in isolated patches across the Himalaya—Hengduan Mountains (including Bhutan, northeast India, Myanmar, Nepal, Sikkim), Southwest, and Central China (Phartyal et al., 2012), whereas *C. cathayanum* mainly occurs in isolated stands of montane deciduous forests in Southeast China. By contrast, *C. cordatum* is native to Japan and certain islands in the Russian Far East (Sakhalin, Kuriles; Araki et al., 2010). All three species of *Cardiocrinum* have self-compatible, visually showy flowers, and are insect (many bumblebee species) pollinated flowers that mature into capsules containing several 100 seeds with thin filmy wings (Ohara et al., 2006). Despite taxonomic recognition of three distinct species within the genus, the possibility of hybridization has long been suspected from morphological and/or distributional considerations, especially between the parapatric species pair *C. giganteum* and *C. cathayanum* with abutting ranges in Central China. In addition, although recent molecular phylogenetic studies supported the monophyly of Lilieae and recovered *Cardiocrinum* spp. as one of the early diverging lineages (Hayashi and Kawano, 2000; Patterson and Givnish, 2002; Gao et al., 2012; Kim et al., 2013), species relationships within *Cardiocrinum* largely remained unclear because usually only *C. giganteum* was included in all previous studies. Therefore, it is necessary to construct a robust phylogenetic tree of *Cardiocrinum* to facilitate a better understanding of the speciation, diversification, and biogeography of the genus in East Asia.

*Cardiocrinum* species are widely grown as ornamental plants in temperate regions of the Northern Hemisphere for their large and gorgeous flowers (Phartyal et al., 2012). On the other hand, they are known to contain bioactive compounds, such as isopimarane-type diterpenoids (Liu, 1984) and inhibitors of 5-lipoxygenase activation, as well as high levels of various trace elements, such as Ca, Mg, Fe, and Zn (Wang et al., 2007). In China, *Cardiocrinum* species are locally used as medicinal plants and food sources. For example, *Cardiocrinum* seeds have been proven to be a potential herbal replacement for *Aristolochia* fruits in treating cough (Li et al., 2010); and the starchy bulbs of *C. giganteum* are the staple food of local people in Guangxi and Yunnan (Li, 1997). The great economic value of *Cardiocrinum* species has brought about overexploitation and habitat fragmentation/isolation of their natural populations (Li et al., 2012), which might decrease not only population size but also genetic diversity. Despite of its ecological and economic importance, molecular research of *Cardiocrinum* has lagged far behind. So far, only a few microsatellite loci have been developed for *C. cordatum* and *C. giganteum* (Abdoullaye et al., 2010; Li et al., 2012). Evidently more effective molecular markers are needed to foster efforts regarding the identification, conservation, utilization, and breeding of *Cardiocrinum* species in the context of phylogeographic and population genetic analyses.

Chloroplasts, derived from photosynthetic bacteria, have their own genomes encoding an array of proteins in relation to photosynthesis, nitrogen fixation and biosynthesis of starch, pigments, fatty acids, and amino acids (Neuhaus and Emes, 2000; Howe et al., 2003; Liu et al., 2012). In contrast to nuclear genomes, plant chloroplast genomes show high copy numbers per cell and a much smaller size for complete sequencing (McNeal et al., 2006). The chloroplast genomes in angiosperms usually have a circular structure ranging from 115 to 165 kb in length and consist of two copies of a large inverted repeat (IR) region separated by a large single-copy (LSC) region and a small single-copy (SSC) region (Raubeson and Jansen, 2005; Wicke et al., 2011; Shetty et al., 2016). Due to the lack of recombination, low rates of nucleotide substitutions, and usually uniparental inheritance, chloroplast DNA sequences are a primary source of data for inferring plant phylogenies (Shaw et al., 2005). With the development of next-generation sequencing (NGS) technology, it is now more convenient to obtain complete chloroplast genome sequences and promptly extend gene-based phylogenetics to phylogenomics. Whole chloroplast genomes are increasingly being used for phylogenetic analyses and have proven to be effective in resolving evolutionary relationships, especially at lower taxonomic levels where recent divergence, and rapid radiations have resulted in limited sequence variation by using traditional methods (Cai et al., 2015; Ruhsam et al., 2015).

Here, we present the complete and annotated DNA sequences for the cp genomes of the three *Cardiocrinum* species. Our study aims were as follows: (1) to investigate global structural patterns of *Cardiocrinum* cp genomes; (2) examine variations of simple sequence repeats (SSRs) and repeat sequences among the three *Cardiocrinum* cp genomes; (3) to evaluate the morphology-based classification of *Cardiocrinum* species and resolve their phylogenetic relationships using the chloroplast genome sequence data; and (4) to screen fast evolving DNA regions among the three chloroplast genomes. The results will provide abundant information for the identification as well as phylogenetic, phylogeographic and population genetic studies of *Cardiocrinum* species, and aid in the conservation and utilization of their genetic resources.

## Materials and methods

### Plant material and DNA extraction

Fresh leaves of *C. giganteum* from Sichuan Province (China), *C. cathayanum* from Zhejiang Province (China), and *C. cordatum* from the Miyazaki Prefecture (Japan) were sampled and dried with silica gel. Voucher specimens were deposited in the Herbarium of Zhejiang University (HZU). Genomic DNA was extracted from approximately 3 mg of the silica-dried leaf tissue using DNA Plantzol Reagent (Invitrogen) according to the manufacturer's protocol. The quality and concentration of the DNA products were assessed using agarose gel electrophoresis and an Agilent BioAnalyzer 2100 (Agilent Technologies).

### DNA sequencing and genome assembly

Purified DNA was used to generate short-insert (500 bp) paired-end sequencing libraries according to the Illumina standard protocol. Genomic DNA from each species was indexed by tags and pooled together in one lane of an HiSeq™ 2000 (Illumina, San Diego, California, USA) for sequencing at Beijing Genomics Institute (BGI, Shenzhen, China). For each species, approximately 2.0 Gb of raw data were generated with pair-end 125 bp read length. The raw reads were assembled into whole chloroplast genomes in a multi-step approach employing a modified pipeline that involved a combination of both reference guided and *de novo* assembly approaches (Cronn et al., 2008). First, paired-end sequence reads were trimmed to remove low-quality bases (*Q* < 20, 0.01 probability error) and adapter sequences using CLC-quality trim tool (quality_trim software included in CLC ASSEMBLY CELL package, http://www.clcbio.com/products/clc-assembly-cell/) before undertaking sequence assembly. Second, the contigs were assembled using CLC *de novo* assembler with the following optimized parameters: bubble size of 98, minimum contig length of 200, mismatch cost of 2, deletion and insertion costs of 3, length fraction of 0.9, and similarity fraction of 0.8. Third, all the contigs were aligned to the reference chloroplast genome of *L. longiflorum* (KC968977) using BLAST (http://blast.ncbi.nlm.nih.gov/), and aligned contigs (≥90% similarity and query coverage) were ordered according to the reference chloroplast genome. Then, contigs were aligned with the reference genome to construct the draft chloroplast genome of each species in Geneious 9.0.5 software (http://www.geneious.com). Finally, clean reads were remapped to the draft genome sequences and yield the complete chloroplast genome sequences.

### Genome annotation and whole genome comparison

The chloroplast genomes were annotated by using the program DOGMA (Dual Organellar GenoMe Annotator; Wyman et al., 2004), coupled with manual corrections for start and stop codons. Protein-coding genes were identified by using the plastid/bacterial genetic code. Intron/exon boundaries were further determined using MAFFT v7 (Katoh and Standley, 2013) with those of the chloroplast genomes of *L. longiflorum* and *Fritillaria hupehensis* Hsiao et K. C. Hsia (NC024736) as references. We also used the program tRNAscan-SE (Schattner et al., 2005) with default settings to verify tRNA boundaries identified by DOGMA. The graphical maps of the *Cardiocrinum* chloroplast genomes were drawn using the OrganellarGenome DRAW tool (ORDRAW; Lohse et al., 2007), with subsequent manual editing.

The mVISTA program (http://genome.lbl.gov/vista/mvista/submit.shtml) was used to compare the complete plastid genome of *C. giganteum* with those of *C. cathayanum* and *C. cordatum*, taking the annotation of the chloroplast genome of *Lilium longiflorum* as a reference. Default parameters were utilized to align the chloroplast genomes in Shuffle-LAGAN mode and a sequence conservation profile was visualized in an mVISTA plot (Frazer et al., 2004). To explore the divergence hotspot regions in *Cardiocrinum* and facilitate its utilization in identification, all the regions, including coding regions, introns and intergenic spacers, were sequentially extracted under the following two criteria: (a) total number of mutation (Eta) > 0; and (b) an aligned length >200 bp. The nucleotide variability was calculated with DnaSP 5.10 (Librado and Rozas, 2009). Any large structural events, such as gene order rearrangements and IR expansions/contractions, were recorded.

### Characterization of repeat sequences and SSRs

Size and location of repeat sequences, including direct (forward), inverted (palindromic), complement, and reverse repeats in the *Cardiocrinum* chloroplast genomes were identified by running REPuter (Kurtz and Schleiermacher, 1999). For all the repeat types, the constraint set in REPuter was 90% or greater sequence identity with hamming distance equal to 3. Simple sequence repeats (SSRs) were detected using MISA perl script (Thiel et al., 2003) with thresholds of 10 repeat units for mononucleotide SSRs, 5 repeat units for dinucleotide SSRs, 4 repeat units for trinucleotide SSRs, and 3 repeat units for tetra-, penta-, and hexa-nucleotide SSRs.

### Phylogenetic analysis

Altogether the complete chloroplast genome sequences of 12 species from Liliaceae were used for phylogenetic analysis (Table S1), including four *Fritillaria* species, four *Lilium* species, *Erythronium sibiricum* (Fisch. & C.A.Mey.) Krylov (P. Li, unpublished data) and the three *Cardiocrinum* species sequenced here (Table S1). Because of the close relationship of Liliaceae and Smilacaceae, *Smilax china* L. of Smilacaceae (Liu et al., 2012) was included as outgroup (Table S1). The sequences were aligned using MAFFT v7 (Katoh and Standley, 2013) and manually edited where necessary. The unambiguously aligned DNA sequences were used for phylogenetic tree construction. In order to examine the phylogenetic utility of different regions, phylogenetic analyses were performed using Maximum likelihood (ML) and Bayesian inference (BI) methods based on the following two data sets: (1) the complete chloroplast genome sequences; and (2) a set of 74 protein-coding genes shared by the chloroplast genomes of the13 species (Table S2). In both analyses, all the gaps were excluded after alignment.

We analyzed the above two data matrices under both ML and BI frameworks using an unpartitioned strategy. In addition, we also conducted partitioned analyses for 74-gene data set using two model partitionin strategies: (1) partitioning by codon position (three partitions), and (2) partitioning by each gene (74 partitions). ML analysis was conducted using RAxML-HPC v8.2.8 with 1000 bootstrap replicates on the CIPRES Science Gateway website (Miller et al., 2010). Akaike Information Criterion (AIC) in jModelTest v2.1.4 (Posada, 2008) was used to determine the best-fitting models of nucleotide substitutions and a GTR + G + I substitution model was selected for both data sets. BI analyses were conducted in MrBayes v3.2 (Ronquist and Huelsenbeck, 2003). The Markov chain Monte Carlo (MCMC) algorithm was run for two million generations with trees sampled every 500 generations. The first 25% of generations were discarded as burn-in. A 50% majority-rule consensus tree was constructed from the remaining trees to estimate posterior probabilities (PPs).

## Results and discussion

### Genome organization and features

**Figure 1:**
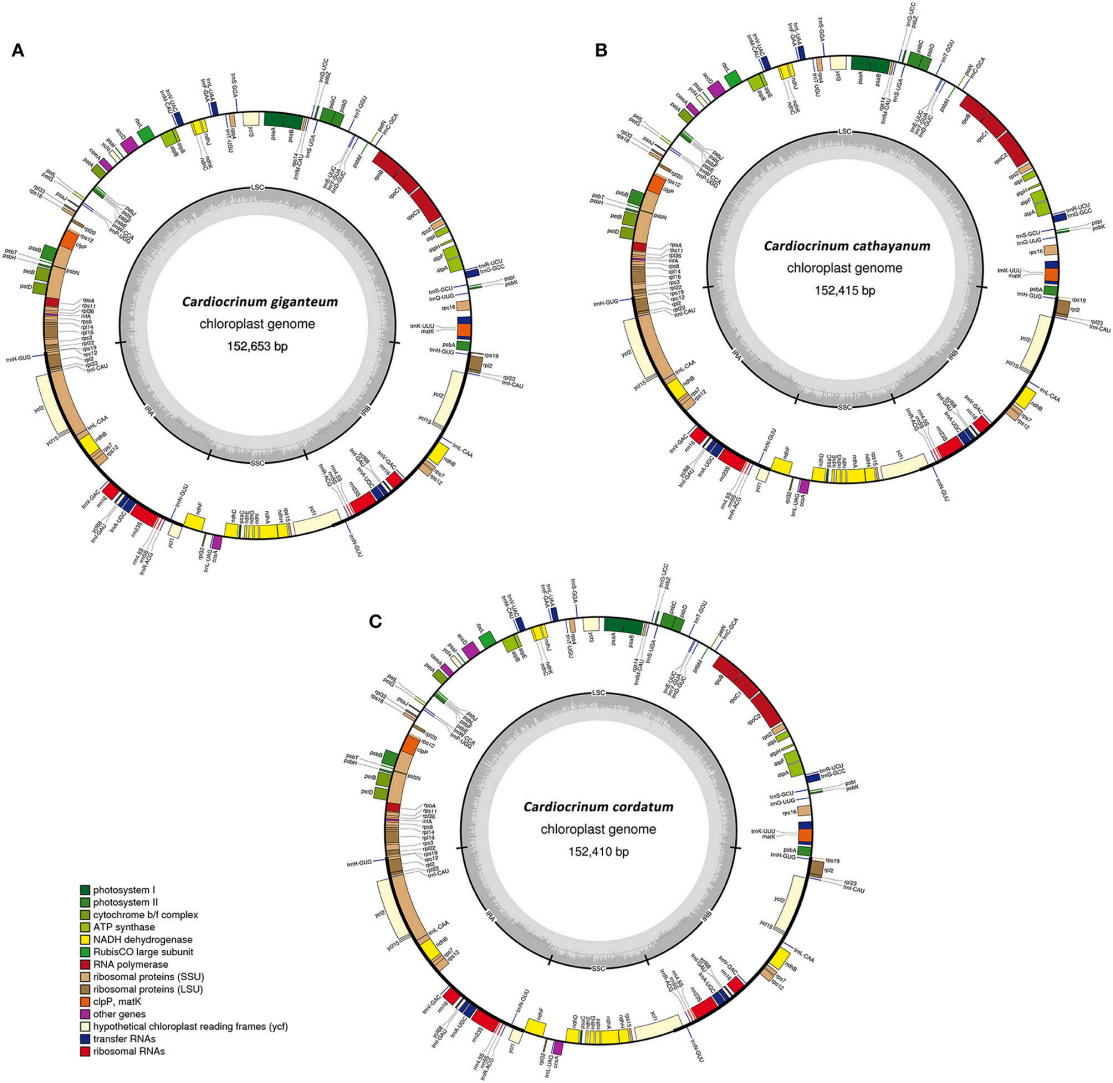
**Gene maps of the three ***Cardiocrinum*** chloroplast genomes. (A)** *Cardiocrinum giganteum*; **(B)** *C. cathayanum*; **(C)** *C. cordatum*. Genes shown on the outside of the circle are transcribed clockwise, and genes inside are transcribed counter-clockwise. Genes belonging to different functional groups are color-coded. The darker gray in the inner corresponds to GC content, and the lighter gray corresponds to AT content.

Illumina paired-end (125 bp) sequencing produced 16,593,274, 17,071,940 and 16,590,680 clean reads for *C. giganteum, C. cathayanum, C. cordatum*, respectively. The *de novo* assembly generated 17,157 contigs with an N50 length of 351 bp and a total length of 6.38 Mb for *C. cathayanum*, 20,859 contigs with an N50 length of 366 bp and a total length of 8.25 Mb for *C. cordatum*, and 26,859 contigs with an N50 length of 391 bp and a total length of 11.55 Mb for *C. giganteum* (Table 1). Each draft chloroplast genome was generated from a combined product of four initial contigs, with no gaps and no Ns. The determined nucleotide sequences of the three *Cardiocrinum* chloroplast genomes ranged narrowly from 152,410 bp in *C. cordatum* to 152,653 bp in *C. giganteum* (Figure 1, Table 1). All three chloroplast genomes exhibited the general quadripartite structure typical of angiosperms, consisting of a pair of IRs (26,364–26,500 bp) separated by the LSC (82,186–82,368 bp) and SSC (17,309–17,319 bp) regions. The chloroplast genome sequences were deposited in GenBank (accession numbers, KX528334 for *C. giganteum*, KX575836 for *C. cathayanum*, and KX575837 for *C. cordatum*).

**Table 1:** **The basic characteristics of three ***Cardiocrinum*** chloroplast genomes**.

**Characteristics**	***C. giganteum***	***C. cathayanum***	***C. cordatum***
Clean reads	16,593,274	17,071,940	16,590,680
Average read length (bp)	125	125	125
Number of contigs	26,859	17,157	20,859
Total length of contigs (bp)	11,547,060	6,383,866	8,245,296
N50 length of contigs (bp)	391	351	366
Total cpDNA size (bp)	152,653	152,415	152,410
LSC length (bp)	82,344	82,368	82,186
SSC length (bp)	17,309	17,319	17,344
IR length (bp)	26,500	26,364	26,440
Total CDS length (bp)	72,870	72,201	72,846
Total tRNA length (bp)	2879	2880	2881
Total rRNA length	9046	9046	9050
Total GC content (%)	37.1	37.1	37.1
LSC	34.9	34.9	34.9
SSC	30.8	30.9	30.9
IR	42.5	42.5	42.5
Total number of genes	132	132	132
Protein-coding genes	78	78	78
rRNAs genes	4	4	4
tRNAs genes	30	30	30
Duplicated genes	20	20	20

The three *Cardiocrinum* chloroplast genomes encoded an identical set of 132 genes, of which 112 were unique and 20 were duplicated in the IR regions (Table 2), and the arrangements of these 132 genes in them were totally collinear. The 112 unique genes included 78 protein-coding genes, 30 tRNA genes, and 4 rRNA genes. Protein-coding regions accounted for 47.37–47.80% of the whole genome, while tRNA and rRNA regions accounted for 1.89 and 5.93–5.94%, respectively (Table 1). The remaining regions were non-coding sequences, including intergenic spacers, introns, and pseudogenes. The overall GC content was 37.1%, whereas the GC content in the LSC, SSC and IR regions were 34.9, 30.8–30.9, and 42.5%, respectively (Table 1), indicating nearly identical levels among the three *Cardiocrinum* chloroplast genomes. The GC content of the *Cardiocrinum* chloroplast genomes is close to that reported in other Liliales chloroplast genomes (Liu et al., 2012; Do et al., 2013; Kim and Kim, 2013).

**Table 2:** **Gene composition of ***Cardiocrinum*** chloroplast genomes**.

**Groups of genes**	**Names of genes**
Ribosomal RNAs	*rrn16(× 2), rrn23(× 2), rrn4.5(× 2), rrn5(× 2)*
	*trnK-UUU*^a^*, trnQ-UUG, trnS-GCU, trnG-GCC*^a^, *trnR-UCU*
	*trnC-GCA, trnD-GUC, trnY-GUA, trnE-UUC, trnT-GGU*
	*trnS-UGA, trnG-UCC, trnfM-CAU, trnS-GGA, trnT-UGU*
Transfer RNAs	*trnL-UAA*^a^*, trnF-GAA, trnV-UAC*^a^*, trnM-CAU, trnW-CCA*
	*trnP-UGG, trnH-GUG(× 2), trnI-CAU(× 2), trnL-CAA(× 2)*
	*trnV-GAC(× 2), trnI-GAU*^a^*(× 2), trnA-UGC*^a^*(× 2), trnR-ACG(× 2)*
	*trnN-GUU(× 2), trnL-UAG*
Photosystem I	*psaB, psaA, psaI, psaJ, psaC*
Photosystem II	*psbA, psbK, psbI, psbM, psbD, psbC, psbZ, psbJ, psbL, psbF, psbE, psbB, psbT, psbN, psbH*
Cytochrome	*petN, petA, petL, petG, petB*^a^*, petD*^a^
ATP synthase	*atpA, atpF*^a^*, atpH, atpI, atpE, atpB*,
Rubisco	*rbcL*
NADH dehydrogenase	*ndhJ, ndhK, ndhC, ndhB*^a^*(× 2), ndhF, ndhD, ndhE*,
	*ndhG, ndhI, ndhA*^a^*, ndhH*
ATP-dependent protease subunit P	*clpP*^b^
Chloroplast envelope membrane protein	*cemA*
large units	*rpl33, rpl20, rpl36, rpl14, rpl16*^a^*, rpl22, rpl2*^a^*(× 2), rpl23(× 2), rpl32*
small units	*rps16*^a^*, rps2, rps14, rps4, rps18, rps12*^b^*(× 2), rps11, rps8, rps3, rps19, rps7(× 2), rps15*
RNA polymerase	*rpoC2, rpoC1*^a^*, rpoB, rpoA*,
Miscellaneous proteins	*matK, accD, ccsA*
Hypothetical proteins & conserved reading frames	*ycf3*^b^*, ycf4, ycf2(× 2), ycf1*
Pseudogenes	*^Ψ^ycf15(× 2), ^Ψ^ycf68(× 2),^Ψ^infA*

a *Indicates the genes containing a single intron*.

b *Indicates the genes containing two introns; (× 2) indicates genes duplicated in the IR regions; pseudogene is represented by ^Ψ^*.

**Figure 2:**
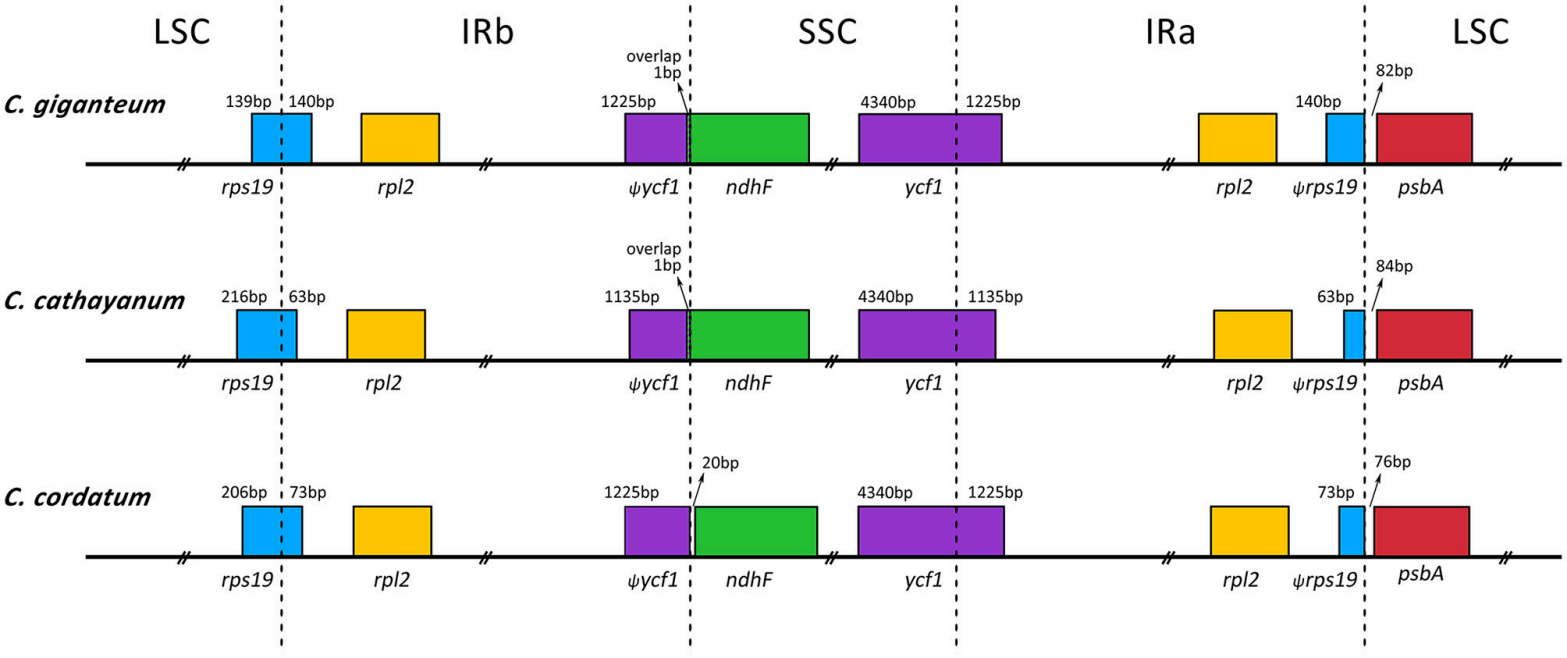
**Comparison of LSC, IR, and SSC junction positions among the three ***Cardiocrinum*** chloroplast genomes**.

Nine of the protein-coding genes and six of the tRNA genes possessed a single intron, whereas three genes (*rps12, clpP*, and *ycf3*) contained two introns (Table 2). All the protein-coding genes had standard AUG as initiator codon. The gene *rps12* was trans-spliced; the 5′ end exon was located in the LSC region and the 3′ exon and intron were duplicated and located in the IR regions. The *infA* region that contained several internal stop codons and is thus interpreted as pseudogenes. The pseudogenization of *infA* is also found in other angiosperm chloroplast genomes (Schmitz-Linneweber et al., 2001; Sloan et al., 2014; Raman and Park, 2015). Whether or not *ycf68* and *ycf15* occur as pseudogenes or protein-coding genes has already been discussed in previous studies (Raubeson et al., 2007). In general, based only on their sequence conservation over broad evolutionary distances and lack of internal stop codons, the two regions (*ycf15* and *ycf68*) have been hypothesized to represent functional protein-coding genes (Raubeson et al., 2007). However, in the present study, they appear as pseudogenes because their coding sequences (CDS) contain several internal stop codons. Thus, the sequences of *ycf15* and *ycf68* are not annotated in the *Cardiocrinum* genomes. Furthermore, the *rps19* gene, located in the boundary region between LSC and IRa, has apparently lost its protein-coding ability due to partial gene duplication. The same phenomenon was also found in the *ycf1* gene at the SSC and IRb border (Figure 2).

### Contraction and expansion of inverted repeats (IRs)

Generally, the lengths of IR (IRa and IRb) regions differ among various plant species. The expansion and contraction of the IR regions and the single-copy (SC) boundary regions often results in length variation of angiosperm chloroplast genomes (Kim and Lee, 2004). We compared exact IR/SC border positions and their adjacent genes among the three *Cardiocrinum* chloroplast genomes (Figure 2). Although overall genomic structure including gene number and gene order was well-conserved, the three *Cardiocrinum* chloroplast genomes exhibited obvious differences at the IR/SC boundary regions (Figure 2). The IR region expanded into the *rps19* gene, creating a pseudogene fragment ψ*rps19* at the IRa/LSC border with lengths of 63–140 bp (*C. giganteum*: 140 bp; *C. cathayanum*: 63 bp; *C. cordatum*: 73 bp). The *ycf1* gene crossed the SSC/IRa region and the pseudogene fragment ψ*ycf1* was located at the IRb region with 1135–1225 bp. For *C. giganteum* and *C. cathayanum* chloroplast genomes, the *ndhF* gene and the ψ*ycf1* fragment overlapped by 1 bp at the junction of the IRa and SSC regions. However, for *C. cordatum*, the *ndhF* gene was entirely located in the SSC region and the distance between *ndhF* and ψ*ycf1* was 20 bp (Figure 2).

### Comparative genomic analysis of the genus cardiocrinum

**Figure 3:**
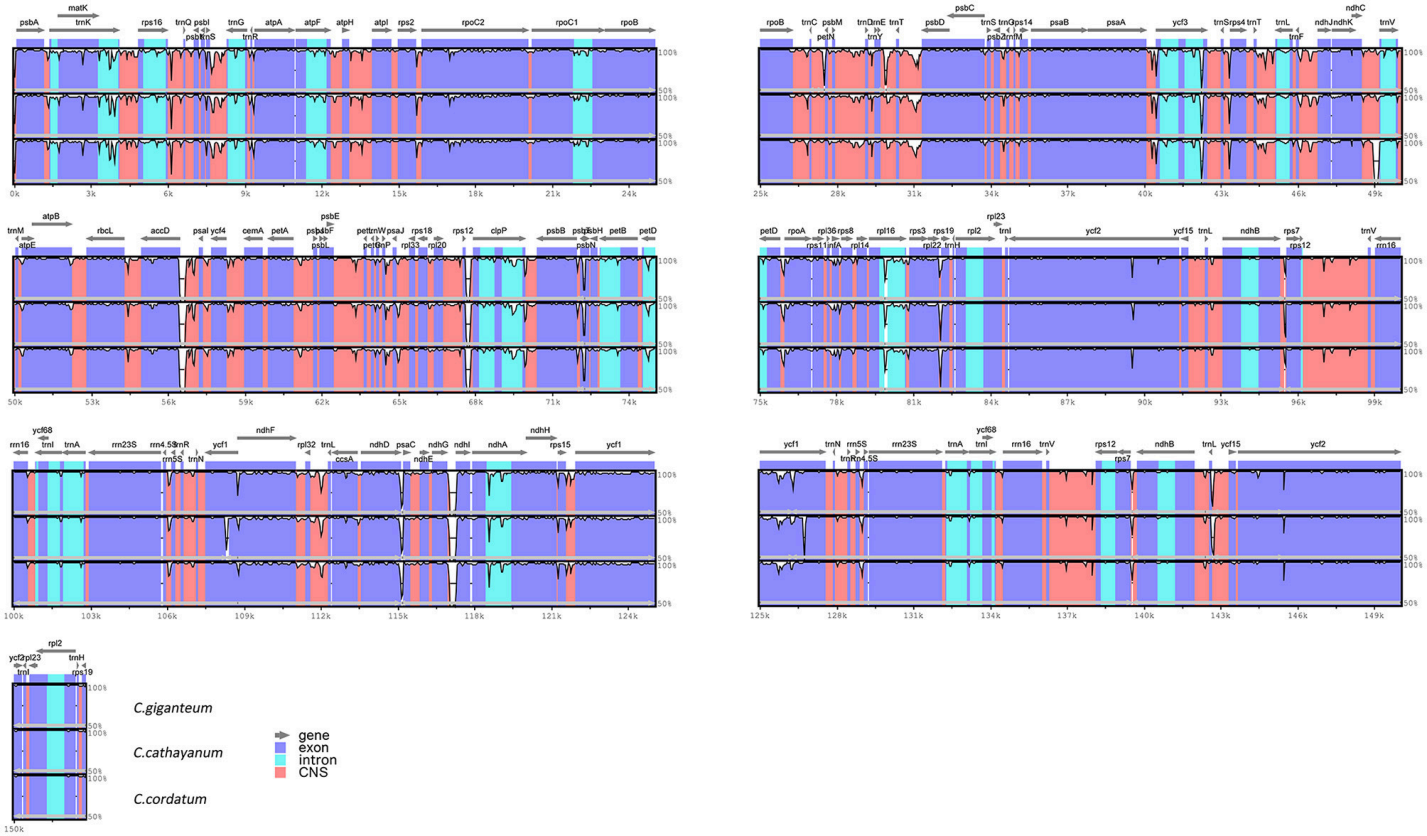
**Sequence identity plots among the three ***Cardiocrinum*** chloroplast genomes, with ***Lilium longiflorum*** as a reference**. Annotated genes are displayed along the top. The vertical scale represents the percent identity between 50 and 100%. Genome regions are color coded as exon, intron, and conserved non-coding sequences (CNS).

**Figure 4:**
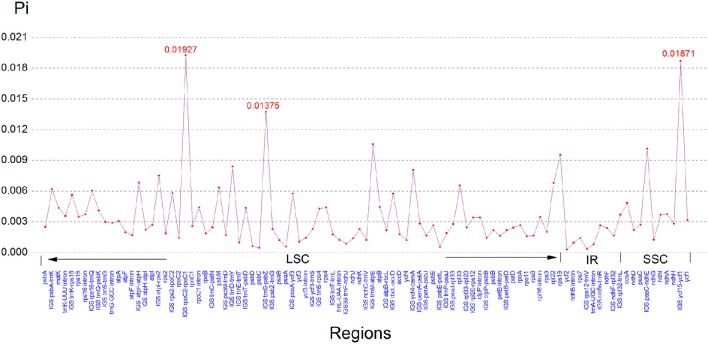
**The nucleotide variability (Pi) values were compared among ***C. giganteum***, ***C. cathayanum*** and ***C. cordatum*****.

Comparison of the sequences revealed several regions of high sequence length polymorphism (Figure 3). Being largely consistent with recent studies (Nazareno et al., 2015; Yao et al., 2015; Zhang et al., 2016), most of the sequence variations were found to be located in the LSC and SSC regions, while the IR regions exhibited comparatively fewer sequence variations. The lower sequence divergence observed in the IRs than SC regions for *Cardiocrinum* species and other angiosperms is likely due to copy correction between IR sequences by gene conversion (Khakhlova and Bock, 2006). We eventually identified 97 regions (43 coding regions, 42 intergenic spacers, and 12 introns) with more than 200 bp in length. Of these 97 regions, nucleotide variability (Pi) ranged from 0.0003 (*ycf2*) to 0.01927 (*rpoC2-rpoC1*) among the three *Cardiocrinum* species (Figure 4; Table S3). As found in most angiosperms (Zhang et al., 2011; Choi et al., 2016), sequence divergence in intergenic regions was higher than that in genic regions of these three chloroplast genomes. The mean value of Pi in non-coding regions was 0.42%, which was almost twice as much as in the coding regions (0.27% on average). Intergenic regions with a percentage of Pi exceeding 1% were *rpoC2-rpoC1, trnS-psbZ, trnM-atpE, psaC-ndhE*, and *ycf15-ycf1*. However, the highest proportion of variability in genic regions was 0.96% (*rps19*) (Figure 4; Table S3). Together, these six divergence hotspot regions should be useful for developing molecular markers for phylogenetic and phylogeographic analyses as well as plant identification of *Cardiocrinum* species.

### Repeat structure and SSR analysis

**Figure 5:**
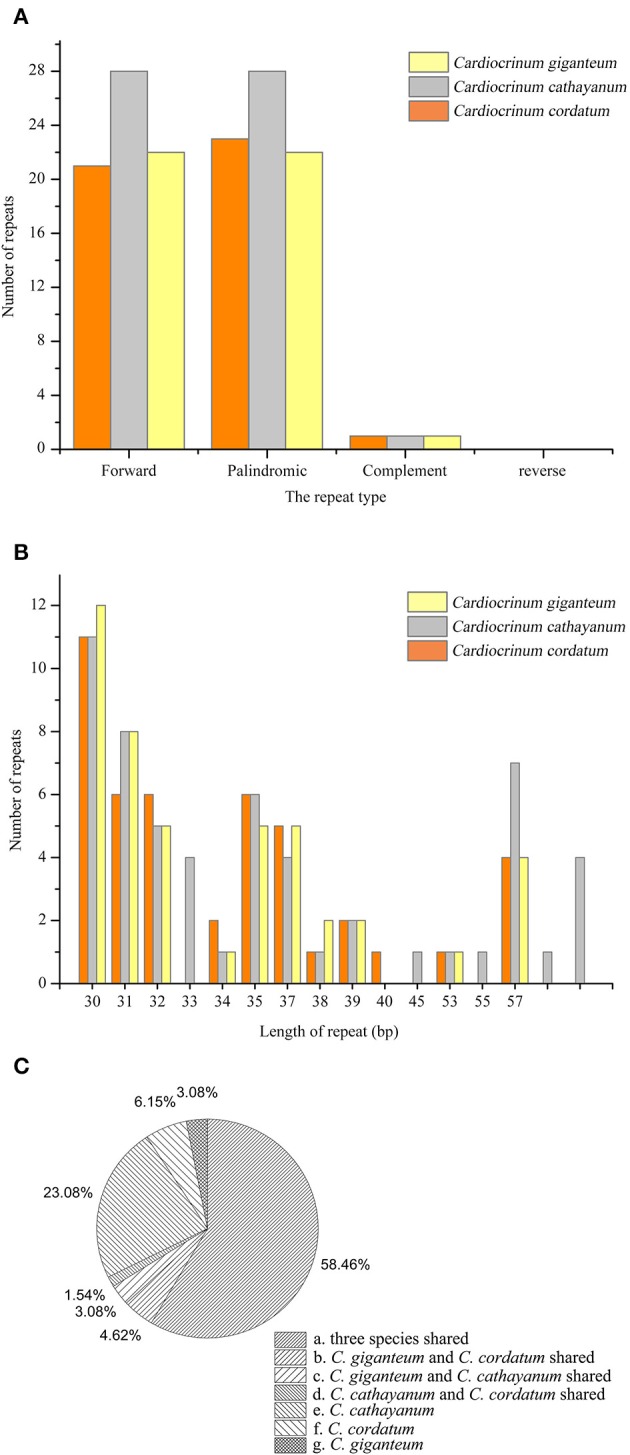
**Analysis of repeated sequences in the three ***Cardiocrinum*** chloroplast genomes. (A)** Frequency of repeats by length; **(B)** Frequency of repeat types; **(C)** Summary of the shared repeats among the *Cardiocrinum* cp genomes.

A total of 147 repeats, including forward, palindromic and complement repeats, were detected in the three *Cardiocrinum* chloroplast genomes using REPuter (Kurtz and Schleiermacher, 1999; Figure 5A; Table S4). *Cardiocrinum cathayanum* contained the most repeats (57) comprising of 28 forward repeats, 28 palindromic repeats, and 1 complement repeat (Table S4). The other two species identically possessed 45 repeats (*C. cordatum*: 21 forward repeats, 23 palindromic repeats and 1 complement repeat; *C. giganteum*: 22 forward repeats, 22 palindromic repeats, and 1 complement repeat). The majority of repeats (88.9%) ranged from 30 to 40 bp in size (Figure 5B; Table S4). Under the criterion with identical lengths located in homologous regions as shared repeats, we investigated those repeats shared among the three *Cardiocrinum* chloroplast genomes. There were 38 repeats shared by the three *Cardiocrinum* chloroplast genomes, 3 repeats shared by *C. giganteum* and *C. catahayanum*; 2 repeats shared by *C. giganteum* and *C. cordatum* and 1 repeat shared by *C. cathayanum* and *C. cordatum*. Additionally, *C. cathayanum* owned the most unique repeats (15) while *C. cordatum* and *C. giganteum* had only four and two unique repeats, respectively (Figure 5C; Table S4). Repeats located in gene *ycf2* occupied 47.7% (31 repeats) of total distinct repeats and 32.3% (21 repeats) were located in non-coding regions, while some were found in genes such as *psaB, rps16, ycf3*, and *trnS-GCU* (Table S4).

**Figure 6:**
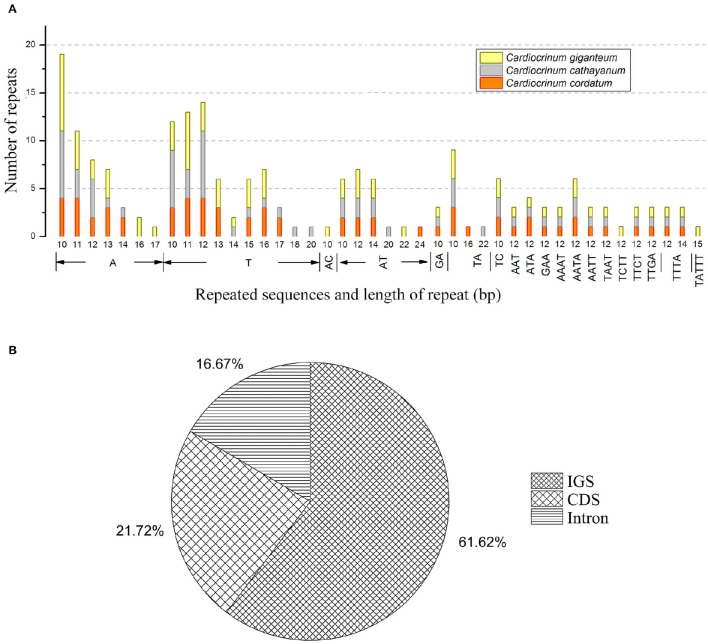
**Simple sequence repeats (SSRs) in the three ***Cardiocrinum*** chloroplast genomes. (A)** Numbers of SSRs by length; **(B)** Distribution of SSR loci. IGS: intergenic spacer region.

SSRs or microsatellites in the chloroplast genome present high diversity in copy numbers, and are important molecular markers for plant population genetics and evolutionary studies (Bodin et al., 2013; Zhao et al., 2015; Zhang et al., 2016). With MISA analysis, each *Cardiocrinum* chloroplast genome was found to contain 63–71 SSRs (*C. giganteum*: 71; *C. cathayanum*: 64; *C. cordatum*: 63) (Figure 6A; Table S5), of which 33 SSRs were the same for the three chloroplast genomes (similar repeat units located in similar genomic regions; Table S6), and the numbers of polymorphic SSRs ranged from 30 to 38. Among these SSRs, the mononucleotide A/T repeat units occupied the highest proportion with 59.2% in *C. giganteum*, 59.4% in *C. cathayanum* and 55.3% in *C. cordatum* (Figure 6A; Table S5). These numbers are slightly lower than those reported in previous studies on asterids (68%) and monocots (76%) (Huotari and Korpelainen, 2012; Qian et al., 2013). Among the total 198 SSRs, most loci were located in intergenic spacer (IGS) regions (60.3%), followed by CDS (23.1%) and introns (16.6%) (Figure 6B). This may be due to the fact there is a higher mutation rate in the IGS regions than the coding regions. We observed that 15 different SSRs were located in 9 protein-coding genes [*ycf1* (× 5), *cemA, rpoC2* (× 3), *ycf2* (× 2), *ndhH, rpl22, ndhD, ndhE, cemA*] of the three *Cardiocrinum* chloroplast genomes. In general, the SSRs of these chloroplast genomes showed abundant variation, and can therefore be used in future population genetic studies of *Cardiocrinum* species.

### Phylogenetic analysis

**Figure 7:**
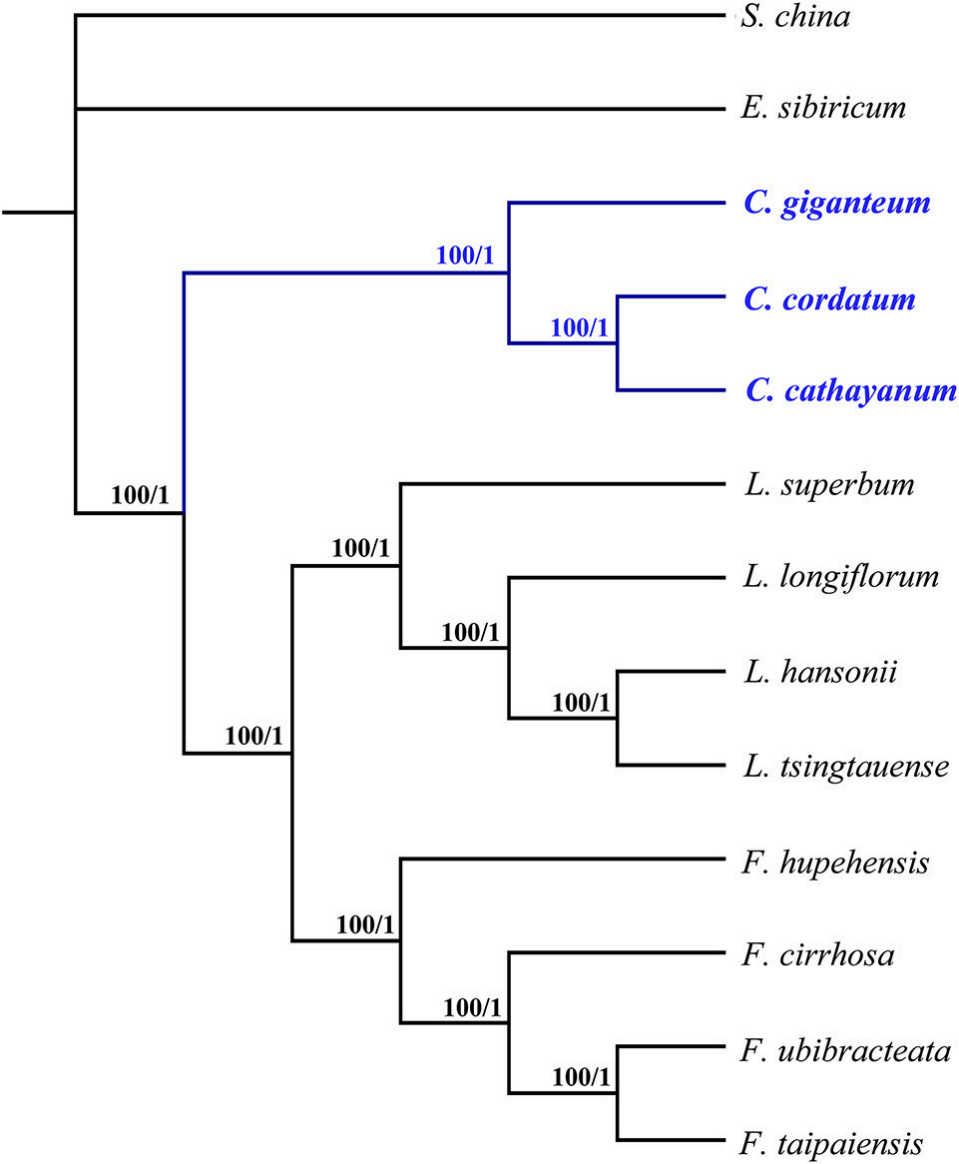
**Phylogenetic relationships of the three ***Cardiocrinum*** species inferred from Maximum likelihood (ML) and Bayesian inference (BI) based on complete genome sequences using no partitioning scheme**. Numbers above the lines represent ML bootstrap values and BI posterior probability. The phylogenetic tree based on 74 protein-coding genes is completely consistent with this topology.

The whole chloroplast genomes and protein-coding genes have been successfully used to resolve phylogenetic relationships at almost any taxonomic level during the past decade (De Las Rivas et al., 2002; Moore et al., 2007; Zhang et al., 2016). In the present study, two data sets including 74 commonly present protein-coding genes (Table S2) and the complete chloroplast genome sequences of 12 species from Liliaceae were used to perform phylogenetic analysis, with *Smilax china* (Smilacaceae) used as outgroup (Table S1). The BI and ML analyses yielded nearly identical tree topologies across all analyses, with 100% bootstrap (BS) values and 1.0 Bayesian posterior probabilities (PP) at each node (Figure 7). Thus, only the phylogenetic trees based on complete genome sequences using no partitioning scheme are shown. All these phylogenetic trees identically supported the monophyly of Cardiocrinum, which in turn formed a sister clade to the *Lilium*+*Fritillaria* group. The phylogenetic trees in this study also indicate a sister relationship of *Fritillaria* to *Lilium*, which is consistent with a previous phylogenetic study based on four plastid loci (Kim et al., 2013). Within *Cardiocrinum, C. giganteum* from the HHM region/Southwest China was identified as sister to *C. cathayanum* (Southeast China)—*C. cordatum* (Japan, Russian Far East Islands; Figure 7). According to Wu and Wu (1998), the Sino-Japanese Floristic Region (SJFR) can be divided into two subkingdoms: the Sino-Himalayan and the Sino-Japanese Forest subkingdoms. The phylogenetic relationships in *Cardiocrinum* are found to be consistent with Wu and Wu (1998) floristic division. Major genetic subdivisions between the Sino-Himalayan and Sino-Japanese Forest subkingdoms have also been found in other plant taxa (e.g., *Spiraea japonica* complex: Zhang et al., 2006; *Ainsliaea*: Mitsui et al., 2008) and likewise across the East China Sea between Southeast China and Japan (e.g., *Kalopanax septemlobus*: Sakaguchi et al., 2012; *Euptelea*: Cao et al., 2016). However, considering that chloroplast genome is a haploid, uniparentally-inherited, single locus (Birky, 1995), comparative phylogenies and phylogeography between biparental (nuclear) and uniparental (chloroplast) markers are needed to elucidate the timing and processes underlying species diversification, hybridization and range evolution within *Cardiocrinum*. Overall, our phylogenomic analyses based on chloroplast genomes have provided the first successful attempt to clarify intrageneric relationships within *Cardiocrinum*. In addition, they also recovered phylogenetic relationships within the tribe Lilieae, which are consistent with previous phylogenetic results based on chloroplast and/or nuclear markers (Gao et al., 2012; Kim et al., 2013).

## Conclusions

In this study, the chloroplast genomes of the three species of *Cardiocrinum* are reported for the first time and their organization is described. These three chloroplast genomes exhibit typical quadripartite and circular structure that is rather conserved in genomic structure and the synteny of gene order. However, these chloroplast genomes show obvious variations at the boundaries of the four regions because of the expansion and contraction of the inverted repeat (IR) regions and the single-copy (SC) boundary regions. The six rapidly evolving regions and 147 repeat sequences identified in the *Cardiocrinum* chloroplast genome can be selected for future studies to develop markers and conduct phylogenetic analysis. In addition, the cp SSRs with abundant variation identified herein should be useful in characterizing the population genetic structure of *Cardiocrinum* species. Our phylogenomic analyses based on two data sets including 13 species from Liliaceae and Smilacaceae provided strong support for the monophyly of *Cardiocrinum* as sister to *Fritillaria*–*Lilium* within the tribe Lilieae. Furthermore, within *Cardiocrinum, C. giganteum* was identified as sister to *C. cathayanum–C. cordatum*, which thus reflects a biogeographically interesting phylogenetic tripartition of the genus across the SJFR. Overall, the data obtained in this study will be beneficial to expand our understanding of the evolutionary history of the tribe Lilieae in general, and the times and modes of *Cardiocrinum* diversification in particular.

## Author contributions

YQ conceived the ideas; RL and PL contributed to the sampling; RL performed the experiment and analyzed the data. The manuscript was written by RL and YQ.

### Conflict of interest statement

The authors declare that the research was conducted in the absence of any commercial or financial relationships that could be construed as a potential conflict of interest.
