# Supplementary material for: The Complete Chloroplast Genomes of Three Cardiocrinum (Liliaceae) Species: Comparative Genomic and Phylogenetic Analyses
Source: Front Plant Sci. 2017 Jan 10;7:2054. doi: 10.3389/fpls.2016.02054 (PMC5222849; doi:10.3389/fpls.2016.02054)
Supplement: Supplementary file 1 [file Table1.DOCX]

Table S1. Accession numbers of chloroplast genomes used for phylogenetic analyses

| Family | Genus | Taxon | GenBank NO. | Source |
| --- | --- | --- | --- | --- |
| Liliaceae | *Cariocrinum* | *Cariocrinum giganteum* | KX528334 | This article |
| Liliaceae | *Cariocrinum* | *Cariocrinum cathayanum* | KX575836 | This article |
| Liliaceae | *Cariocrinum* | *Cariocrinum cordatum* | KX575837 | This article |
| Liliaceae | *Erythronium* | *Erythronium sibiricum* | KX644899 | Unpublished data |
| Liliaceae | *Lilium* | *Lilium superbum* | NC026787 | Mennes et al., 2015 |
| Liliaceae | *Lilium* | *Lilium hansonii* | KM103364 | Unpublished data |
| Liliaceae | *Lilium* | *Lilium tsingtauense* | KM103365 | Unpublished data |
| Liliaceae | *Lilium* | *Lilium longiflorum* | KC968977 | Kim and Kim, 2013 |
| Liliaceae | *Fritillaria* | *Fritillaria unibracteata* var. *wabuensis* | KF769142 | Li et al., 2016 |
| Liliaceae | *Fritillaria* | *Fritillaria cirrhosa* | NC024728 | Li et al., 2014 |
| Liliaceae | *Fritillaria* | *Fritillaria hupehensis* | NC024736 | Li et al., 2014 |
| Liliaceae | *Fritillaria* | *Fritillaria taipaiensis* | KF769144 | Li et al., 2014 |
| Smilacaceae | *Smilax* | *Smilax china* | HM536959 | Liu et al., 2012 |
