# Supplementary material for: The Complete Chloroplast Genomes of Three Cardiocrinum (Liliaceae) Species: Comparative Genomic and Phylogenetic Analyses
Source: Front Plant Sci. 2017 Jan 10;7:2054. doi: 10.3389/fpls.2016.02054 (PMC5222849; doi:10.3389/fpls.2016.02054)
Supplement: Supplementary file 2 [file Table2.DOCX]

Table S2. The names of 74 protein-coding genes used for phylogenetic analyses

| *gene name* | *gene neme* | *gene name* | *gene name* |
| --- | --- | --- | --- |
| *rpl2* | *rpl16* | *psbF* | *psb2* |
| *rpl23* | *rpl14* | *psbL* | *psbD* |
| *ycf2* | *rps8* | *psbJ* | *psbC* |
| *ndhB* | *rpl36* | *petL* | *psbM* |
| *rps15* | *rps11* | *petJ* | *petN* |
| *ndhH* | *rpoA* | *petA* | *rpoB* |
| *ndhA* | *petD* | *ycf4* | *rpoC1* |
| *ndhI* | *petB* | *psaI* | *rpoC2* |
| *ndhG* | *psbH* | *rbcL* | *rps2* |
| *ndhE* | *psbN* | *atpB* | *atpI* |
| *psaC* | *psbT* | *atpE* | *atpH* |
| *ndhD* | *psbB* | *ndhC* | *atpF* |
| *ccsA* | *rps12* | *ndhK* | *atpA* |
| *rpl32* | *rpl20* | *ndhJ* | *psbK* |
| *ndhF* | *rps18* | *rps4* | *rps16* |
| *rps7* | *rpl33* | *ycf3* | *matK* |
| *rps19* | *petG* | *psaA* | *psbA* |
| *rpl22* | *pet2* | *psaB* |  |
| *rps3* | *psbE* | *rps14* |  |
