# Supplementary material for: The Complete Chloroplast Genomes of Three Cardiocrinum (Liliaceae) Species: Comparative Genomic and Phylogenetic Analyses
Source: Front Plant Sci. 2017 Jan 10;7:2054. doi: 10.3389/fpls.2016.02054 (PMC5222849; doi:10.3389/fpls.2016.02054)
Supplement: Supplementary file 3 [file Table3.DOCX]

Table S3. Nucleotide variability (Pi) values and total number of mutation (Eta) in *Cardiocrinum*

| Regions | Pi | Eta | Regions | Pi | Eta |
| --- | --- | --- | --- | --- | --- |
| *psbA* | 0.00251 | 4 | IGS *trnM-atpE* | 0.01058 | 3 |
| IGS *psbA-trnK* | 0.0062 | 2 | *atpB* | 0.00445 | 10 |
| *matK* | 0.00437 | 10 | IGS *atpB-rbcL* | 0.00219 | 2 |
| *trnK-UUU* intron | 0.00358 | 14 | IGS *rbcL-accD* | 0.00577 | 6 |
| IGS *trnK-rps16* | 0.00563 | 6 | *accD* | 0.0018 | 4 |
| *rps16* | 0.0035 | 6 | *ycf4* | 0.0012 | 1 |
| *rps16* intron | 0.00374 | 5 | IGS *ycf4-cemA* | 0.00805 | 6 |
| IGS *rps16-trnQ* | 0.00604 | 7 | IGS *cemA-petA* | 0.00282 | 1 |
| IGS *trnQ-psbK* | 0.0041 | 2 | IGS *petA-psbJ* | 0.00167 | 3 |
| IGS *trnS-trnG* | 0.00299 | 3 | *psbE* | 0.00265 | 1 |
| *trnG-GCC* intron | 0.00289 | 3 | IGS *psbE-petL* | 0.00056 | 1 |
| *atpA* | 0.00306 | 7 | IGS *trnP-psaJ* | 0.00189 | 1 |
| *atpF* | 0.00199 | 4 | IGS *psaJ-rpl33* | 0.00278 | 2 |
| *atpF* intron | 0.0017 | 2 | *rpl33* | 0.00654 | 2 |
| IGS *atpF-atpH* | 0.00682 | 5 | IGS *rpl33-rpl20* | 0.00247 | 1 |
| IGS *atpH-atpI* | 0.00225 | 3 | IGS *rpl20-rps12* | 0.00343 | 4 |
| *atpI* | 0.00269 | 3 | *clpP* intron | 0.00343 | 7 |
| IGS *atpI-rps2* | 0.00752 | 3 | IGS *clpP-psbB* | 0.00144 | 1 |
| *rps2* | 0.00188 | 2 | *psbB* | 0.00218 | 5 |
| IGS *rps2-rpoC2* | 0.0058 | 2 | *petB* intron | 0.00165 | 2 |
| *rpoC2* | 0.00145 | 9 | IGS *petB-petD* | 0.00217 | 1 |
| IGS *rpoC2-rpoC1* | 0.01927 | 5 | *petD* | 0.00244 | 2 |
| *rpoC1* | 0.0026 | 11 | *rpoA* | 0.00266 | 4 |
| *rpoC1* intron | 0.00441 | 5 | *rps11* | 0.0016 | 1 |
| *rpoB* | 0.00187 | 9 | *rps8* | 0.00167 | 1 |
| IGS *trnC-petN* | 0.00246 | 2 | *rpl16* intron | 0.00347 | 5 |
| *psbM* | 0.00635 | 1 | *rps3* | 0.00203 | 2 |
| IGS *psbM-trnD* | 0.00171 | 3 | *rpl22* | 0.00679 | 4 |
| IGS *trnD-trnY* | 0.00839 | 4 | *rps19* | 0.00956 | 4 |
| IGS *trnE-trnT* | 0.001 | 1 | *ycf2* | 0.0003 | 3 |
| IGS *trnT-psbD* | 0.00436 | 7 | *ndhB* intron | 0.00098 | 1 |
| *psbD* | 0.00063 | 1 | *rps7* | 0.00142 | 1 |
| *psbC* | 0.00047 | 1 | IGS *rps12-trnV* | 0.00036 | 1 |
| IGS *trnS-psbZ* | 0.01375 | 4 | *trnA-UGC* intron | 0.00082 | 1 |
| IGS *psbZ-trnG* | 0.00229 | 1 | IGS *rrn5s-trnR* | 0.00266 | 1 |
| *psaB* | 0.00121 | 4 | *ndhF* | 0.00239 | 8 |
| *psaA* | 0.00059 | 2 | IGS *ndhF-rpl32* | 0.00167 | 1 |
| IGS *psaA-ycf3* | 0.00576 | 5 | IGS *rpl32-trnL* | 0.0037 | 4 |
| *ycf3* | 0.00106 | 3 | *ccsA* | 0.00483 | 7 |
| *ycf3* intron | 0.00145 | 3 | *ndhD* | 0.00221 | 5 |
| IGS *ycf3-trnS* | 0.0023 | 2 | *psaC* | 0.00271 | 1 |
| IGS *trnS-rps4* | 0.00429 | 2 | IGS *psaC-ndhE* | 0.01015 | 6 |
| *rps4* | 0.0044 | 4 | *ndhG* | 0.00125 | 1 |
| IGS *trnT-trnL* | 0.00178 | 2 | *ndhI* | 0.0037 | 3 |
| *trnL-UAA* intron | 0.00124 | 1 | *ndhA* | 0.00375 | 12 |
| IGS *trnF-ndhJ* | 0.00087 | 1 | *ndhH* | 0.00282 | 5 |
| *ndhJ* | 0.0014 | 1 | IGS *ycf15-ycf1* | 0.01871 | 8 |
| *ndhK* | 0.00228 | 3 | *ycf1* | 0.00317 | 26 |
| IGS *ndhC-trnV* | 0.00123 | 1 |  |  |  |

* IGS: Intergenic spacer region; Eta: total number of mutation
