# Supplementary material for: The Complete Chloroplast Genomes of Three Cardiocrinum (Liliaceae) Species: Comparative Genomic and Phylogenetic Analyses
Source: Front Plant Sci. 2017 Jan 10;7:2054. doi: 10.3389/fpls.2016.02054 (PMC5222849; doi:10.3389/fpls.2016.02054)
Supplement: Supplementary file 4 [file Table4.DOCX]

Table S4. Analyses of repeat sequences in the three *Cardiocrinum* chloroplast genomes

| *C. giganteum* | | | *C. cathayanum* | | | *C. cordatum* | | | Location |
| --- | --- | --- | --- | --- | --- | --- | --- | --- | --- |
| L | T | S | L | T | S | L | T | S |  |
|  |  |  | 79 | F | 89576 |  |  |  | *ycf2* |
|  |  |  | 79 | F | 145104 |  |  |  | *ycf2* |
|  |  |  | 64 | F | 145119 |  |  |  | *ycf2* |
| 57 | F | 89632 | 57 | F | 89579 | 57 | F | 89407 | *ycf2* |
| 57 | F | 145284 | 57 | F | 145099 | 57 | F | 145108 | *ycf2* |
|  |  |  | 57 | F | 89603 |  |  |  | *ycf2* |
|  |  |  | 55 | F | 145128 |  |  |  | *ycf2* |
| 53 | F | 37242 | 53 | F | 37286 | 53 | F | 37275 | *psaB* |
|  |  |  | 45 | F | 6094 |  |  |  | IGS/*rps12-trnQ* |
|  |  |  |  |  |  | 40 | F | 6084 | IGS/*rps13-trnQ* |
| 39 | F | 42121 | 39 | F | 42172 | 39 | F | 42161 | *ycf3* |
| 38 | F | 7545 |  |  |  |  |  |  | IGS/*psbI-trnS* |
| 37 | F | 145304 | 37 | F | 89603 | 37 | F | 89407 | *ycf2* |
| 37 | F | 89632 | 37 | F | 145119 | 37 | F | 145104 | *ycf2* |
| 37 | F | 145280 |  |  |  | 37 | F | 145128 | *ycf2* |
| 35 | F | 92503 | 35 | F | 92480 | 35 | F | 92284 | IGS/*ycf15-trnL* |
| 35 | F | 42124 | 35 | F | 42175 | 35 | F | 42164 | *ycf3* |
|  |  |  |  |  |  | 34 | F | 6101 | IGS/*rps13-trnQ* |
|  |  |  | 33 | F | 89579 |  |  |  | *ycf2* |
|  |  |  | 33 | F | 145099 |  |  |  | *ycf2* |
| 32 | F | 89661 | 32 | F | 89632 | 32 | F | 89436 | *ycf2* |
|  |  |  |  |  |  | 32 | F | 108606 | *ycf1* pseudogene |
| 31 | F | 6053 | 31 | F | 6094 |  |  |  | IGS/*rps16-trnQ* |
| 31 | F | 7624 | 31 | F | 7655 | 31 | F | 7654 | *trnS-GCU* |
| 31 | F | 29440 | 31 | F | 29509 |  |  |  | IGS/*trnD-trnY* |
| 31 | F | 145313 | 31 | F | 145128 | 31 | F | 145137 | *ycf2* |
| 30 | F | 3799 |  |  |  | 30 | F | 3796 | IGS/*trnK-UUU* |
| 30 | F | 9122 | 30 | F | 9178 | 30 | F | 9177 | *trnG-GCC* |
| 30 | F | 29443 | 30 | F | 29509 |  |  |  | IGS/*trnD-trnY* |
| 30 | F | 29857 | 30 | F | 29924 | 30 | F | 29915 | IGS/*trnE-trnT* |
| 30 | F | 37268 | 30 | F | 37312 | 30 | F | 37301 | *psaB* |
| 30 | F | 87292 | 30 | F | 87239 | 30 | F | 87067 | *ycf2* |
|  |  |  | 30 | F | 145157 |  |  |  | *ycf2* |
| 30 | F | 147657 | 30 | F | 147496 | 30 | F | 147481 | *ycf2* |
|  |  |  | 79 | P | 89576 |  |  |  | *ycf2* |
|  |  |  | 79 | P | 89600 |  |  |  | *ycf2* |
| 57 | P | 89632 | 57 | P | 89579 | 57 | P | 89407 | *ycf2* |
| 57 | P | 89656 | 57 | P | 89603 | 57 | P | 89431 | *ycf2* |
|  |  |  | 57 | P | 89627 |  |  |  | *ycf2* |
|  |  |  | 57 | P | 89627 |  |  |  | *ycf2* |
| 39 | P | 42121 | 39 | P | 42172 | 39 | P | 42161 | *ycf3* |
| 38 | P | 27574 | 38 | P | 27644 | 38 | P | 27645 | IGS/*trnC-petN* |
| 37 | P | 89632 | 37 | P | 89603 | 37 | P | 89407 | *ycf2* |
| 37 | P | 89680 | 37 | P | 89651 | 37 | P | 89455 | *ycf2* |
|  |  |  | 35 | P | 34830 | 35 | P | 34819 | IGS/*psbZ-trnG* |
| 35 | P | 42124 | 35 | P | 42175 | 35 | P | 42164 | *ycf3* |
| 35 | P | 92503 | 35 | P | 92480 | 35 | P | 92284 | IGS/*ycf15-trnL* |
| 35 | P | 142459 | 35 | P | 142268 | 35 | P | 142277 | IGS/*trnL-CAA* |
| 34 | P | 113109 | 34 | P | 113002 | 34 | P | 112921 | *ccsA* |
|  |  |  | 33 | P | 89579 |  |  |  | *ycf2* |
|  |  |  | 33 | P | 89651 |  |  |  | *ycf2* |
| 32 | P | 34205 | 32 | P | 34240 | 32 | P | 34229 | *trnS-UGA* |
| 32 | P | 89661 | 32 | P | 89632 | 32 | P | 89436 | *ycf2* |
| 32 | P | 89685 | 32 | P | 89656 | 32 | P | 89460 | *ycf2* |
| 32 | P | 113530 | 32 | P | 113423 | 32 | P | 113342 | IGS/*ccsA-ndhD* |
| 31 | P | 7626 | 31 | P | 7657 | 31 | P | 7656 | *trnS-GCU* |
| 31 | P | 76005 | 31 | P | 76031 | 31 | P | 75855 | IGS/*petD-rpoA* |
| 31 | P | 121550 | 31 | P | 121441 | 31 | P | 121361 | IGS/*rps15-ycf1* |
|  |  |  |  |  |  | 30 | P | 3802 | IGS/*trnK-UUU* |
| 30 | P | 5415 | 30 | P | 5456 | 30 | P | 5446 | *rps16* |
| 30 | P | 34816 |  |  |  |  |  |  | IGS/*psbZ-trnG* |
| 30 | P | 57087 | 30 | P | 57122 | 30 | P | 56944 | IGS/*accD-psaI* |
| 30 | P | 87292 | 30 | P | 87239 | 30 | P | 87067 | *ycf2* |
| 30 | P | 87313 | 30 | P | 87260 | 30 | P | 87088 | *ycf2* |
| 31 | C | 44912 | 31 | C | 44960 | 31 | C | 44950 | IGS/*trnT -trnL* |

* L: Repeat length; T: Repeat types; S: start position; F: Forward; P: Palindrome C: Complement
