# Supplementary material for: The Complete Chloroplast Genomes of Three Cardiocrinum (Liliaceae) Species: Comparative Genomic and Phylogenetic Analyses
Source: Front Plant Sci. 2017 Jan 10;7:2054. doi: 10.3389/fpls.2016.02054 (PMC5222849; doi:10.3389/fpls.2016.02054)
Supplement: Supplementary file 5 [file Table5.DOCX]

| Table S5. The simple sequence repeats in *C. giganteum,* *C. cathayanum* and *C. cordatum* | | | | | | | | | | | | | | |
| --- | --- | --- | --- | --- | --- | --- | --- | --- | --- | --- | --- | --- | --- | --- |
| *C. giganteum* | | | | | *C. cathayanum* | | | | | *C. cordatum* | | | | |
| Repeat unit | No. repeat unit | Start position | End position | Location | Repeat unit | No. repeat unit | Start position | End position | Location | Repeat unit | No. repeat unit | Start position | End position | Location |
| A | 10 | 6213 | 6222 | IGS | A | 10 | 6607 | 6616 | IGS | A | 10 | 66326 | 66335 | IGS |
|  |  | 6553 | 6562 | IGS |  |  | 28575 | 28690 | IGS |  |  | 73005 | 73014 | Intron |
|  |  | 13542 | 13551 | IGS |  |  | 29480 | 29541 | IGS |  |  | 120930 | 120939 | IGS |
|  |  | 30609 | 30618 | IGS |  |  | 43747 | 43756 | IGS |  |  | 125336 | 125345 | CDS (ycf1) |
|  |  | 45491 | 45500 | IGS |  |  | 45531 | 45540 | IGS |  | 11 | 13231 | 13241 | IGS |
|  |  | 65251 | 65260 | IGS |  |  | 73180 | 73189 | Intron |  |  | 30644 | 30654 | IGS |
|  |  | 73154 | 73163 | Intron |  |  | 125417 | 125426 | CDS (ycf1) |  |  | 43736 | 43746 | IGS |
|  |  | 125519 | 125528 | CDS (ycf1) |  | 11 | 13233 | 13243 | IGS |  |  | 65106 | 65116 | IGS |
|  | 11 | 4060 | 4070 | IGS |  |  | 65284 | 65294 | IGS |  | 12 | 7351 | 7362 | IGS |
|  |  | 29444 | 29454 | IGS |  |  | 111052 | 111062 | IGS |  |  | 29505 | 29516 | IGS |
|  |  | 31480 | 31490 | IGS |  | 12 | 4095 | 4106 | IGS |  | 13 | 4083 | 4095 | IGS |
|  |  | 59255 | 59265 | CDS (cemA) |  |  | 27001 | 27012 | IGS |  |  | 27002 | 27014 | IGS |
|  | 12 | 13170 | 13181 | IGS |  |  | 45334 | 45345 | IGS |  |  | 110967 | 110979 | IGS |
|  |  | 45280 | 45291 | IGS |  |  | 121008 | 121019 | IGS |  | 14 | 12264 | 12277 | Intron |
|  | 13 | 43696 | 43708 | IGS |  | 13 | 12267 | 12279 | Intron |  |  | 45324 | 45337 | IGS |
|  |  | 77867 | 77879 | IGS |  | 14 | 30652 | 30665 | IGS | T | 10 | 1667 | 1676 | Intron |
|  |  | 82185 | 82197 | IGS | T | 10 | 15854 | 15877 | IGS |  |  | 50628 | 50637 | IGS |
|  | 16 | 7294 | 7310 | IGS |  |  | 28085 | 28094 | IGS |  |  | 121968 | 121977 | CDS (ycf1) |
|  |  | 12197 | 12212 | Intron |  |  | 29480 | 29541 | IGS |  | 11 | 18060 | 18070 | CDS (rpoC2) |
|  | 17 | 111157 | 111172 | IGS |  |  | 69591 | 69600 | Intron |  |  | 29474 | 29484 | IGS |
| T | 10 | 9379 | 9388 | IGS |  |  | 111926 | 111935 | IGS |  |  | 69415 | 69425 | Intron |
|  |  | 35466 | 35475 | IGS |  |  | 122049 | 122058 | CDS (ycf1) |  |  | 122808 | 122818 | CDS (ycf1) |
|  |  | 122151 | 122160 | CDS (ycf1) |  | 11 | 17949 | 18068 | CDS (rpoC2) |  | 12 | 15862 | 15873 | IGS |
|  | 11 | 63601 | 63611 | IGS |  |  | 30338 | 30348 | IGS |  |  | 17951 | 17962 | CDS (rpoC2) |
|  |  | 15808 | 15818 | IGS |  |  | 122889 | 122899 | CDS (ycf1) |  |  | 30329 | 30340 | IGS |
|  |  | 17999 | 18009 | CDS (rpoC2) |  | 12 | 1681 | 1692 | Intron |  |  | 40514 | 40525 | IGS |
|  |  | 28015 | 28025 | IGS |  |  | 4300 | 4311 | IGS |  | 13 | 4289 | 4301 | IGS |
|  |  | 68848 | 68858 | Intron |  |  | 15854 | 15877 | IGS |  |  | 63454 | 63466 | IGS |
|  |  | 122990 | 123000 | CDS (ycf1) |  |  | 17949 | 18068 | CDS (rpoC2) |  |  | 121464 | 121476 | IGS |
|  | 12 | 4265 | 4276 | IGS |  |  | 40525 | 40536 | IGS |  | 15 | 68699 | 68713 | Intron |
|  |  | 17890 | 17901 | CDS (rpoC2) |  |  | 63633 | 63644 | IGS |  |  | 111843 | 111857 | IGS |
|  |  | 80879 | 80890 | IGS |  |  | 80897 | 80908 | IGS |  | 16 | 22520 | 22535 | Intron |
|  | 13 | 1673 | 1685 | Intron |  | 14 | 141544 | 121557 | IGS |  |  | 80721 | 80736 | IGS |
|  |  | 13312 | 13324 | IGS |  | 15 | 68875 | 68889 | Intron |  |  | 123251 | 123266 | CDS (ycf1) |
|  |  | 121653 | 121665 | IGS |  | 16 | 123332 | 123347 | CDS (ycf1) |  | 17 | 9433 | 9449 | IGS |
|  | 14 | 112036 | 112049 | IGS |  | 17 | 22518 | 22534 | Intron |  |  | 122595 | 122611 | CDS (ycf1) |
|  | 15 | 29410 | 29475 | IGS |  | 18 | 9435 | 9452 | IGS | AT | 5 | 30039 | 30048 | IGS |
|  |  | 30291 | 30305 | IGS |  | 20 | 122676 | 122695 | CDS (ycf1) |  |  | 74272 | 74281 | IGS |
|  |  | 69560 | 69574 | Intron | AT | 5 | 30048 | 30057 | IGS |  | 6 | 31161 | 31172 | IGS |
|  | 16 | 40481 | 40496 | IGS |  |  | 74448 | 74457 | IGS |  |  | 75887 | 75898 | IGS |
|  |  | 122778 | 122793 | CDS (ycf1) |  | 6 | 31172 | 31183 | IGS |  | 7 | 7877 | 7890 | IGS |
|  |  | 123433 | 123448 | CDS (ycf1) |  |  | 76063 | 76074 | IGS |  |  | 46019 | 46032 | Intron |
| AC | 5 | 7310 | 7319 | IGS |  | 7 | 7878 | 7891 | IGS |  | 12 | 3806 | 3829 | Intron |
| AT | 5 | 30001 | 30010 | IGS |  |  | 46028 | 46041 | Intron | GA | 5 | 87690 | 87699 | CDS (ycf2) |
|  |  | 74422 | 74431 | IGS |  | 10 | 3822 | 3841 | Intron | TA | 5 | 19426 | 19435 | CDS (rpoC2) |
|  | 6 | 3817 | 3828 | Intron | GA | 5 | 87862 | 87871 | CDS (ycf2) |  |  | 26666 | 26675 | IGS |
|  |  | 31126 | 31137 | IGS | TA | 5 | 19424 | 19433 | CDS (rpoC2) |  |  | 46611 | 46620 | IGS |
|  |  | 76037 | 76048 | IGS |  |  | 26665 | 26674 | IGS |  | 8 | 29517 | 29532 | IGS |
|  | 7 | 7821 | 7834 | IGS |  |  | 46615 | 46624 | IGS | TC | 5 | 120437 | 120446 | CDS (ndhH) |
|  |  | 45988 | 46001 | Intron |  | 11 | 29480 | 29541 | IGS |  |  | 146898 | 146907 | CDS (ycf2) |
|  | 11 | 29454 | 29475 | IGS | TC | 5 | 120518 | 120527 | CDS (ndhH) | AAT | 4 | 41391 | 41402 | Intron |
| GA | 5 | 87915 | 87924 | CDS (ycf2) |  |  | 146913 | 146922 | CDS (ycf2) | ATA | 4 | 6096 | 6107 | IGS |
| TA | 5 | 19365 | 19374 | CDS (rpoC2) | AAT | 4 | 41402 | 41413 | Intron |  |  | 6144 | 6155 | IGS |
|  |  | 26605 | 26614 | IGS | ATA | 4 | 6148 | 6159 | IGS | GAA | 4 | 57161 | 57172 | IGS |
|  |  | 46575 | 46584 | IGS | GAA | 4 | 57339 | 57350 | IGS | AAAT | 3 | 116803 | 116814 | IGS |
| TC | 5 | 120625 | 120634 | CDS (ndhH) | AAAT | 3 | 116884 | 116895 | IGS | AATA | 3 | 81643 | 81654 | CDS (rpl22) |
|  |  | 147074 | 147083 | CDS (ycf2) | AATA | 3 | 81815 | 81826 | CDS (rpl22) |  |  | 113681 | 113692 | CDS (ndhD) |
| AAT | 4 | 41351 | 41362 | Intron |  |  | 113762 | 113773 | CDS (ndhD) | AATT | 3 | 15082 | 15093 | IGS |
| ATA | 4 | 6093 | 6104 | IGS | AATT | 3 | 15084 | 15095 | IGS | TAAT | 3 | 28678 | 28689 | IGS |
| GAA | 4 | 57304 | 57315 | IGS | TAAT | 3 | 28575 | 28690 | IGS | TTCT | 3 | 80335 | 80346 | Intron |
| AAAT | 3 | 116992 | 117003 | IGS | TTCT | 3 | 80511 | 80522 | Intron | TTGA | 3 | 115768 | 115779 | CDS (ndhE) |
| AATA | 3 | 81797 | 81808 | CDS (rpl22) | TTGA | 3 | 115849 | 115860 | CDS (ndhE) | TTTA | 3 | 22088 | 22103 | Intron |
|  |  | 113869 | 113880 | CDS (ndhD) | TTTA | 3 | 40823 | 40834 | IGS |  | 4 | 40812 | 40823 | IGS |
| AATT | 3 | 15027 | 15038 | IGS |  | 4 | 22086 | 22101 | Intron |  |  |  |  |  |
| TAAT | 3 | 28609 | 28620 | IGS |  |  |  |  |  |  |  |  |  |  |
| TCTT | 3 | 12424 | 12435 | IGS |  |  |  |  |  |  |  |  |  |  |
| TTCT | 3 | 80493 | 80504 | Intron |  |  |  |  |  |  |  |  |  |  |
| TTGA | 3 | 115956 | 115967 | CDS (ndhE) |  |  |  |  |  |  |  |  |  |  |
| TTTA | 3 | 40782 | 40793 | IGS |  |  |  |  |  |  |  |  |  |  |
|  | 4 | 22027 | 22042 | Intron |  |  |  |  |  |  |  |  |  |  |
| TATTT | 3 | 22457 | 22471 | Intron |  |  |  |  |  |  |  |  |  |  |
