# Supplementary material for: The Complete Chloroplast Genomes of Three Cardiocrinum (Liliaceae) Species: Comparative Genomic and Phylogenetic Analyses
Source: Front Plant Sci. 2017 Jan 10;7:2054. doi: 10.3389/fpls.2016.02054 (PMC5222849; doi:10.3389/fpls.2016.02054)
Supplement: Supplementary file 6 [file Table6.DOCX]

Table S6. SSRs shared by the three *Cardiocrinum* chloroplast genomes

|  |  | *C. giganteum* | | *C. cathayanum* | | *C. cordatum* | |  |
| --- | --- | --- | --- | --- | --- | --- | --- | --- |
| Repeat unit | No.repaet unit | Start position | End position | Start position | End position | Start position | End position | location |
| A | 10 | 73154 | 73163 | 73180 | 73189 | 73005 | 73014 | intron |
|  |  | 125519 | 125528 | 125417 | 125426 | 125336 | 125345 | ycf1 |
| T | 10 | 122151 | 122160 | 122049 | 122058 | 121968 | 121977 | ycf1 |
|  | 11 | 17999 | 18009 | 17949 | 18068 | 18060 | 18070 | rpoC2 |
|  |  | 28015 | 28025 | 30338 | 30348 | 29474 | 29484 | IGS |
|  |  | 122990 | 123000 | 122889 | 122899 | 122808 | 122818 | ycf1 |
|  | 12 | 17890 | 17901 | 17949 | 18068 | 17951 | 17962 | rpoC2 |
|  | 15 | 69560 | 69574 | 68875 | 68889 | 68699 | 68713 | intron |
|  | 16 | 123433 | 123448 | 123332 | 123347 | 123251 | 123266 | ycf1 |
| AT | 5 | 30001 | 30010 | 30048 | 30057 | 30039 | 30048 | IGS |
|  |  | 74422 | 74431 | 74448 | 74457 | 74272 | 74281 | IGS |
|  | 6 | 31126 | 31137 | 31172 | 31183 | 31161 | 31172 | IGS |
|  |  | 76037 | 76048 | 76063 | 76074 | 75887 | 75898 | IGS |
|  | 7 | 7821 | 7834 | 7878 | 7891 | 7877 | 7890 | IGS |
|  |  | 45988 | 46001 | 46028 | 46041 | 46019 | 46032 | Intron |
| GA | 5 | 87915 | 87924 | 87862 | 87871 | 87690 | 87699 | CDS (ycf2) |
| TA | 5 | 19365 | 19374 | 19424 | 19433 | 19426 | 19435 | CDS (rpoC2) |
|  |  | 26605 | 26614 | 26665 | 26674 | 26666 | 26675 | IGS |
|  |  | 46575 | 46584 | 46615 | 46624 | 46611 | 46620 | IGS |
| TC | 5 | 120625 | 120634 | 120518 | 120527 | 120437 | 120446 | CDS (ndhH) |
|  |  | 147074 | 147083 | 146913 | 146922 | 146898 | 146907 | CDS (ycf2) |
| AAT | 4 | 41351 | 41362 | 41402 | 41413 | 41391 | 41402 | Intron |
| ATA | 4 | 6093 | 6104 | 6148 | 6159 | 6144 | 6155 | IGS |
| GAA | 4 | 57304 | 57315 | 57339 | 57350 | 57161 | 57172 | IGS |
| AAAT | 3 | 116992 | 117003 | 116884 | 116895 | 116803 | 116814 | IGS |
| AATA | 3 | 81797 | 81808 | 81815 | 81826 | 81643 | 81654 | CDS (rpl22) |
|  |  | 113869 | 113880 | 113762 | 113773 | 113681 | 113692 | CDS (ndhD) |
| AATT | 3 | 15027 | 15038 | 15084 | 15095 | 15082 | 15093 | IGS |
| TAAT | 3 | 28609 | 28620 | 28575 | 28690 | 28678 | 28689 | IGS |
| TTCT | 3 | 80493 | 80504 | 80511 | 80522 | 80335 | 80346 | Intron |
| TTGA | 3 | 115956 | 115967 | 115849 | 115860 | 115768 | 115779 | CDS (ndhE) |
| TTTA | 3 | 40782 | 40793 | 40823 | 40834 | 22088 | 22103 | IGS |
|  | 4 | 22027 | 22042 | 22086 | 22101 | 40812 | 40823 | Intron |
